# Biosynthesis of macrolactam antibiotics with β-amino acid polyketide starter units

**DOI:** 10.1038/s41429-024-00742-2

**Published:** 2024-05-30

**Authors:** Fumitaka Kudo

**Affiliations:** Department of Chemistry, Tokyo Institute of Technology, 2-12-1 O-okayama, Meguro-ku, Tokyo 152-8551 Japan

**Keywords:** Enzyme mechanisms, Multienzyme complexes

## Abstract

Macrolactam antibiotics incorporating β-amino acid polyketide starter units, isolated primarily from *Actinomycetes* species, show significant biological activities. This review provides a detailed analysis into the biosynthetic studies of vicenistatin, a macrolactam antibiotic with a 3-aminoisobutyrate starter unit, as well as biosynthetic research on related macrolactam compounds. Firstly, the elucidation of a common mechanism for the incorporation of β-amino acid starter units into the polyketide synthase (PKS) is described. Secondly, the unique biosynthetic mechanisms of the β-amino acids that are used to supply the main macrolactam biosynthetic pathways with starter units are discussed. Thirdly, some distinctive post-PKS modification mechanisms that complete macrolactam antibiotic biosynthesis are summarized. Finally, future directions for creating new macrolactam compounds through engineered biosynthesis pathways are described.

## Introduction

**Fig. 1:**
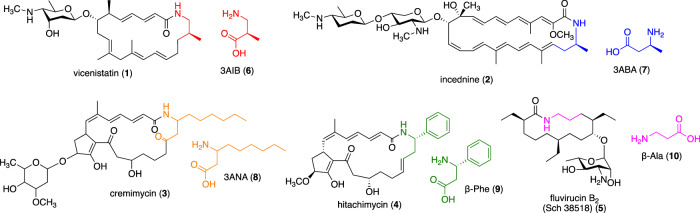
Macrolactam antibiotics studied in our laboratory

Macrolactam antibiotics that incorporate β-amino acids as polyketide starter units have been isolated mostly from the *Actinomycetes* species. These compounds exhibit significant biological activities, including antibacterial, antifungal, and antitumor effects [1]. Vicenistatin (**1**) [2], incednine (**2**) [3], cremimycin (**3**) [4], hitachimycin (stubomycin) (**4**) [5, 6], and fluvirucin B_2_ (Sch 38518, **5**) [7, 8] are examples of such macrolactam antibiotics that have been selected as research targets in our laboratory (Fig. 1); however, many other related macrolactam compounds have also been discovered [9–11]. Vicenistatin (**1**) features 3-aminoisobutylate (3AIB, **6**) as the polyketide starter unit. Similarly, incednine (**2**) has 3-aminobutyrate (3ABA, **7**), cremimycin (**3**) has 3-aminononanoate (3ANA, **8**), hitachimycin (**4**) has β-phenylalanine (β-Phe, **9**), and fluvirucin B2 (**5**) has β-alanine (β-Ala, **10**). The polyketide chain elongates from the β-amino acid starter unit and cyclizes between the β-amino group of the starter unit and the carboxylate moiety of the final extension product, yielding the corresponding macrolactam. As such, this class of macrolactams are biosynthesized via the typical polyketide pathway but with the incorporation of the unique β-amino acid starter units.

**Fig. 2:**
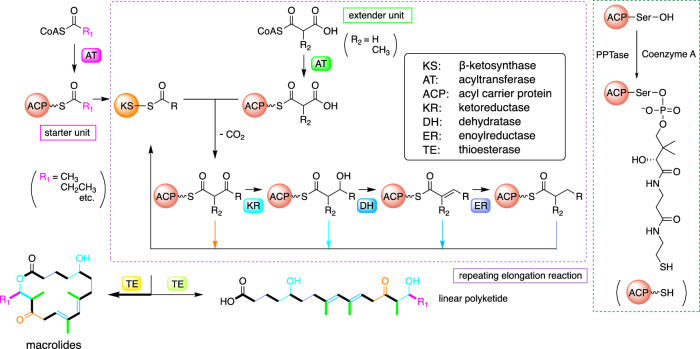
General mechanism of type I polyketide synthase (PKS) for macrocyclic polyketides in bacteria

The biosynthesis of macrocyclic polyketides, and especially that of macrolactones, has been extensively studied [12–14] (Fig. 2). In general, acetate or propionate serves as the starter unit, and malonate and/or methylmalonate act as the extender units to construct the macrocyclic polyketide skeleton. The extender units typically exist as coenzyme A (CoA) thioesters, such as malonyl-CoA and methylmalonyl-CoA, which are transferred to the acyl carrier protein (ACP) domain of the polyketide synthase (PKS) by an acyltransferase (AT) domain, yielding malonyl-ACP/methylmalonyl-ACP [15]. Similarly, starter units are ligated to the ACP, forming acyl-ACP, and there are several methods to achieve this ligation [16]. The starter acyl-ACP is recognized by the β-ketosynthase (KS) domain. The acyl group is transferred to a cysteine residue of the active site of the KS domain and condensed with the extender malonyl-ACP/methylmalonyl-ACP to produce β-ketoacyl-ACP with the release of carbon dioxide. The β-carbonyl group of β-ketoacyl-ACP is subsequently reduced by the β-ketoreductase (KR) domain to yield β-hydroxyacyl-ACP, which is further processed by the dehydratase (DH) domain to generate α,β-unsaturated acyl-ACP. Finally, the enoyl reductase (ER) domain reduces α,β-unsaturated acyl-ACP to provide a fully reduced acyl-ACP.

The second round of polyketide chain elongation is catalyzed by a different set of catalytic domains to extend the chain by one acetate unit. The number of rounds of extension determine the length of the corresponding polyketide and each round is catalyzed by a PKS module consisting of the essential catalytic domains: AT, ACP and KS. The degree of reduction of the polyketide chain is determined by the particular combination of additional tailoring domains (KR, DH, ER), within the module. For example, if the KR domain is absent, the β-carbonyl group remains in the polyketide chain; if the DH domain is absent, the β-hydroxy group remains; and if the ER domain is absent, an olefinic moiety remains. Finally, the thioesterase (TE) domain, located in the terminal PKS module, catalyzes an acyl transfer from the final thioester of the ACP-bound polyketide chain to form an acyl-TE complex, subsequently facilitating lactonization with a hydroxyl group on the elongated polyketide yielding a macrolactone. Post-PKS modifications, including polyketide skeletal modification, oxidation (hydroxylation, epoxidation), glycosylation, methylation, and acylation, are required to complete the biosynthesis of the dead-end polyketide compound [17].

In the biosynthesis of macrolactam antibiotics, the aforementioned PKS reaction is used to construct the polyketide skeleton; however, unique nitrogen-containing starter units are employed. There are several key points of interest regarding β-amino acid starter units. Firstly, their mechanism of incorporation into the PKS machinery. Secondly, as most β-amino acids are non-proteinogenic, their biosynthetic mechanisms are presumably unique. Lastly, the post-PKS modification of macrolactams appears crucial for their biological activities.

This review provides a detailed summary of the biosynthetic studies of vicenistatin, covering the entire biosynthetic pathway. A common mechanism for the incorporation of β-amino acids into the PKS machinery is outlined, emphasizing that adenylation enzymes, selective for β-amino acids, act as gatekeepers, thereby determining incorporation of the unique β-amino acid starter units. Next, the unique mechanisms of β-amino acid biosynthesis and post-PKS modification are described. Finally, future perspectives for creating new molecules based on these biosynthetic studies are discussed.

## Vicenistatin: a macrolactam antibiotic with a β-amino acid starter unit

Vicenistatin (**1**) is a macrolactam antibiotic with 3-aminoisobutyric acid (3AIB, **6**) as the starter unit of the polyketide backbone [2]. Vicenistatin (**1**) and its congener, vicenistatin M, are produced by *Streptomyces halstedii* HC34 [18]. Recently, *Streptomyces parvus* SCSIO Mla-L010 was also found to produce vicenistatin [19]. Vicenistatin exhibits antitumor activity against Co-3 human colon carcinoma cells [2].

**Fig. 3:**
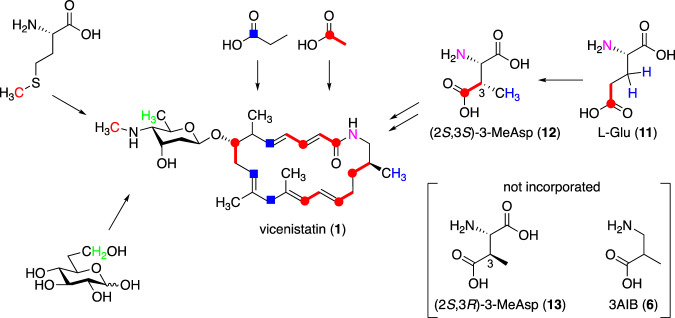
Incorporation studies to investigate the origins of vicenistatin

3AIB (**6**) is a non-proteinogenic β-amino acid with a unique biosynthetic mechanism. Initially, it was unclear how 3AIB (**6**) was incorporated into the polyketide pathway, however, extensive feeding experiments with candidate amino acids have elucidated these mechanisms [20–22]. Critically, l-glutamic acid (**11**) and (2*S*,3*S*)-3-methylaspartic acid (3-MeAsp, **12**) are incorporated into vicenistatin (Fig. 3) by the biosynthetic machinery, but 3AIB (**6**) and (2*S*,3*R*)-3-MeAsp (**13**) are not. Therefore, C–C bond rearrangement of l-Glu (**11**) results in the formation of (2*S*,3*S*)-3-MeAsp (**12**), presumably catalyzed by adenosylcobalamin (AdoCbl) and pyridoxal 5*’*-phosphate (PLP)-dependent glutamate mutase [23]. Decarboxylation of **12** likely occurs after it is incorporated into the polyketide biosynthetic pathway, probably as a thioester derivative with CoA or ACP. Furthermore, epimerization at the C-3 position of **12** most likely occurs during biosynthesis. The other carbons of the aglycon of vicenistatin, called vicenilactam (**14**), are derived from the typical polyketide pathway with malonyl-CoA and methylmalonyl-CoA, as evidenced by feeding experiments with ^13^C-labeled acetate and propionate [20].

The biosynthetic gene cluster (BGC) of vicenistatin was identified using probes targeting NDP-glucose 4,6-dehydratase and NDP-4-keto-6-deoxyglucose 2,3-dehydratase genes [24]. The vicenistatin BGC comprises typical type I PKS genes, deoxyaminosugar biosynthetic enzyme genes, a glutamate mutase gene and putative enzyme genes responsible for incorporating (2*S*,3*S*)-3-MeAsp (**12**) into the polyketide pathway.

**Fig. 4:**
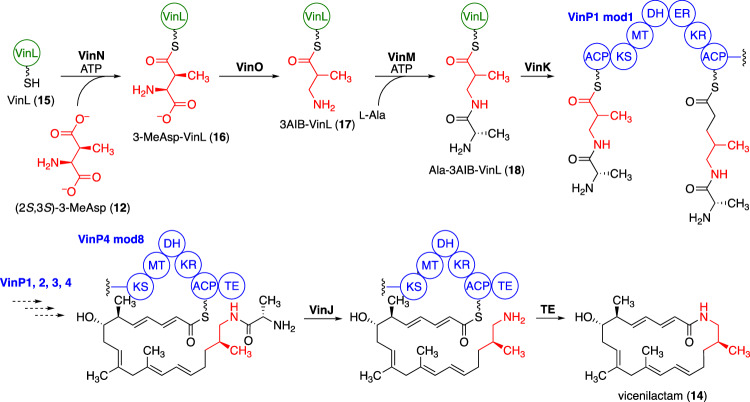
Incorporation mechanism of 3-MeAsp into the polyketide synthase (PKS) in vicenistatin biosynthesis

Functional analysis of the candidate enzymes revealed that an adenylation enzyme, VinN, specifically recognizes 3-MeAsp (**12**), but not 3AIB (**6**), and binds it with a standalone ACP, VinL (**15**), in the presence of ATP to form 3-MeAsp-VinL (**16**) (Fig. 4) [25]. A PLP-dependent enzyme, VinO, then catalyzes the decarboxylation of 3-MeAsp-VinL (**16**) to produce 3AIB-VinL (**17**) [25]. Therefore, it is clear that thioester formation of 3-MeAsp is required before decarboxylation. VinO may not only recognize the 3-MeAsp moiety, but also the ACP moiety VinL. Additionally, epimerization at C-3 presumably occurs during decarboxylation by VinO, yielding (*R*)-3AIB-VinL (**17**), although this has not been clarified. Another adenylation enzyme, VinM, is encoded in the vicenistatin BGC and was found to activate l-Ala, l-Ser, and Gly, but not 3-MeAsp. Moreover, it was discovered that 3AIB-VinL (**17**) serves as an acceptor, generating l-Ala-3AIB-VinL (**18**) [25]. VinM selectively recognizes 3AIB-VinL (**17**), distinguishing it from VinL (**15**) and 3-MeAsp-VinL (**16**). The generated l-Ala-3AIB-VinL (**18**) is recognized by an acyltransferase, VinK, and the dipeptide group is transferred to the *N*-terminal ACP domain of the loading module (Ld-ACP) of PKS VinP1 (Fig. 4) [25].

**Fig. 5:**
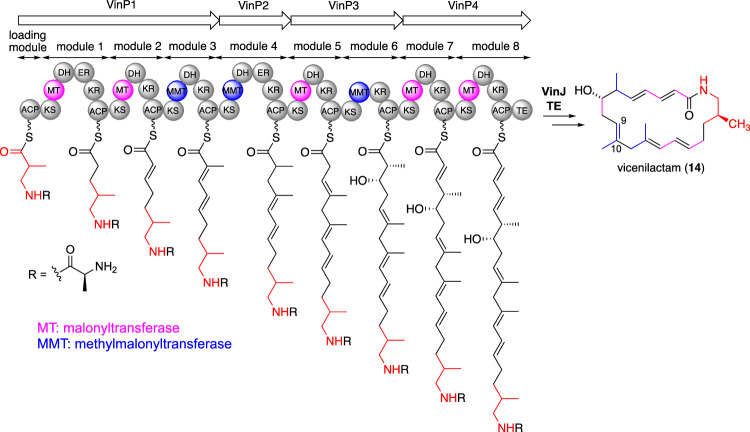
PKS for vicenilactam in vicenistatin biosynthesis

Vicenistatin polyketide synthase (PKS) comprises four typical type I PKSs, VinP1, VinP2, VinP3, and VinP4, and is likely responsible for vicenilactam formation (Fig. 5) [24]. The PKS domain structure corresponds well to the chemical structure of vicenilactam (**14**), including specificity for the extender unit and the degree of reduction at the β-position. However, the mechanism of the C9–C10 double-bond formation remains unclear. The DH domain in module 5 of VinP5 is presumably involved in a unique dehydration process that generates the corresponding trisubstituted olefin. Ultimately, the *C*-terminal thioesterase domain of the VinP4 PKS is responsible for macrolactamization, resulting in the formation of vicenilactam (**14**) [26, 27].

Prior to macrolactamization, an amidohydrolase, VinJ, removes the terminal alanyl moiety from the elongated polyketide chain (Fig. 4) [25, 28]. VinJ appears to recognize the elongated polyketide chain with l-Ala at its terminus, although the exact substrate of recognition and timing of which VinJ recognizes, remain unclear. We propose that the attachment of l-Ala to form l-Ala-3AIB-VinL (**18**) is likely a protective step, blocking the nucleophilic amino group in 3AIB during polyketide elongation (the thermodynamically preferable six-membered lactam can form after the first polyketide extension step if the β-amino group is free; however, the terminal amino group of l-Ala in the dipeptide intermediate cannot access the thioester moiety on the ACP domain of PKS due to the rigid conformation of the amide bond). Thus, the removal of l-Ala from the elongated polyketide intermediate appears to be a deprotection step, generating the nucleophilic amino group derived from 3AIB. This protection–deprotection logic resembles the methodology employed in organic synthesis. Nature seems to favor this type of methodology for the efficient biosynthesis of natural products; for example, amidohydrolase-mediated cleavage reactions are employed in the selective biosynthesis of other dead-end natural products, such as desertomycin [29] and butirosin [30].

**Fig. 6:**
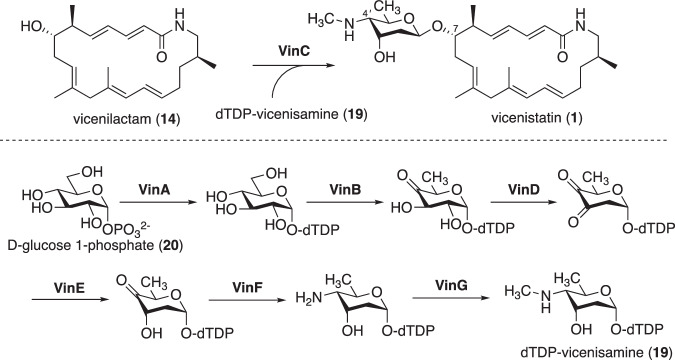
Post-PKS modification in vicenistatin biosynthesis

A glycosyltransferase, VinC, is responsible for attaching the unique deoxyaminosugar, vicenisamine, to complete the biosynthesis [24]. The substrate, dTDP-vicenisamine (**19**), is biosynthesized from d-glucose 1-phosphate (**20**) through the concerted action of the following enzymes: dTDP-glucose synthase VinA, dTDP-glucose 4,6-dehydratase VinB, dTDP-4-keto-6-deoxyglucose 2,3-dehydratase VinD, dTDP-3,4-diketo-2,6-dideoxyglucose 3-ketoreductase VinE, aminotransferase VinF, and *N*-methyltransferase VinG (Fig. 6) [19, 24]. Knocking out the *N*-methyltransferase gene resulted in the production of 4*’*-*N*-demethylvicenistatin, which exhibited potent antibacterial activities [19].

## Incorporation of β-amino acids into the polyketide synthase: a common mechanism

A series of biosynthetic studies on vicenistatin revealed a common logic regarding the mechanism of β-amino acid starter unit incorporation into the polyketide pathway (Fig. 4) [31]. First, a β-amino acid-selective adenylation enzyme activates a pathway-specific β-amino acid and ligates it to a standalone ACP, yielding β-aminoacyl-ACP. Second, another adenylation enzyme activates l-Ala, l-Ser, or Gly, and catalyzes an amide bond-forming reaction with β-aminoacyl-ACP to give dipeptidyl-ACP, presumably for protection of the β-amino group. Third, dipeptidyl-ACP is recognized by a dipeptidyltransferase and transferred to the loading ACP domain at the start of the initial PKS module. Finally, the terminal aminoacyl group is removed by an amidohydrolase prior to macrolactam formation. We have identified the BGCs for incednine (**2**) [32], cremimycin (**3**) [33], hitachimycin (**4**) [34], and fluvircin B_2_ (**5**) [35]; these studies indicated that homologous enzymes are encoded in all BGCs. Thus, this biosynthetic logic is commonly utilized in the biosynthesis of macrolactam antibiotics with β-amino acid starter units. The existence of five homologous enzymes, including a standalone ACP, highlights the potential for BGC identification corresponding to other macrolactam antibiotics that incorporate β-amino acid starter units [9, 10]. An important detail in the biosynthesis of vicenistatin and fluvircin B_2_ is that a decarboxylation reaction (removal of the β-carboxy group from β-aminoacyl-ACP) must occur before the second aminoacylation to give dipeptidyl-ACP.

**Fig. 7:**
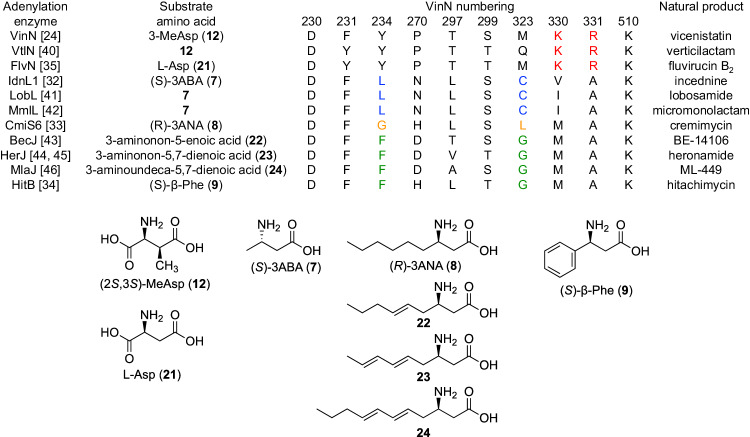
Non-ribosomal (amino acid specificity-conferring) codes for representative β-amino acid selective adenylation enzymes. Unique amino acid residues conserved in each family of enzymes are highlighted in color

Among the five common homologous biosynthetic enzymes, the β-amino acid-selective adenylation enzymes are unique to each biosynthetic pathway [31, 36]. β-Amino acid-selective adenylation enzymes can be classified based on their substrate specificity. These enzymes can recognize β-amino acids with a carboxy group at the β-position, such as 3-MeAsp (**12**) by VinN [24] /VtlN [37] and Asp (**21**) by FlvN [35], a methyl group at the β-position (3-aminobutylic acid, 3ABA, **7**) by IdnL1 [32]/LobL [38]/MmlL [39], a medium-chain alkyl group at the β-position, such as 3-aminononanoic acid (3ANA, **8**) by CmiS6 [33]/BecJ [40]/HerJ [41, 42]/MlaJ [43], and a phenyl group at the β-position (β-phenylalanine, **9**) by HitB [34] (Fig. 7). Crystal structure analysis of VinN [44], CmiS6 [45], IdnL1 [45], and HitB [46] revealed the mechanism of selective recognition of β-amino acids. It is now possible to predict the substrate specificity of β-amino acid-selective adenylation enzymes by comparing their amino acid sequences, particularly for the enzymes involved in this class of macrolactam biosynthesis.

**Fig. 8:**
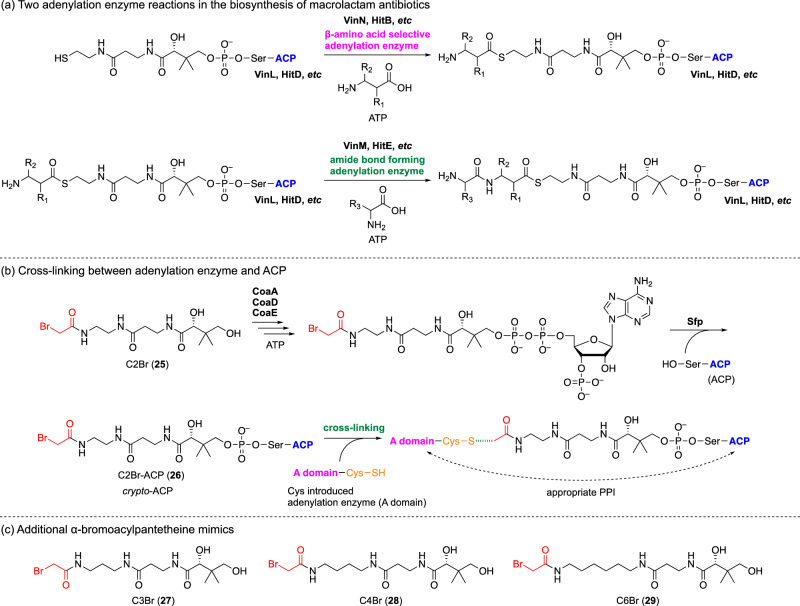
Cross-linking reaction of adenylation enzyme (A domain) and *crypto*-ACP

It is also intriguing how these β-amino acid-selective adenylation enzymes recognize their cognate standalone ACPs (Fig. 8). To elucidate the mechanism by which β-amino acid-selective adenylation enzymes recognize standalone ACPs, we constructed a cross-linked complex using HitB and HitD in hitachimycin biosynthesis [47]. In this system, the original cysteamine moiety in pantetheine is replaced with 1,2-ethylenediamine and further acylated with α-bromoacetic acid, to yield the α-bromoacetamide-containing pantetheine mimic C2Br (**25**). This analog provides an electrophilic functional group for the cross-linking reaction. C2Br (**25**) is enzymatically converted to the corresponding acylated CoA mimic by the CoA biosynthetic enzymes CoaA, CoaD, and CoaE [48]. A promiscuous phosphopantetheinyl transferase, Sfp [49], is then used to transfer the C2Br mimic to the apo form of a standalone ACP to give C2Br-ACP (**26**) as *crypto*-ACP. Next, we mutated an aspartate residue, which is conserved among adenylation enzymes (shown here as A domain), to cysteine. In the wild-type enzyme, the aspartate residue interacts with the amino group of the β-amino acid substrate. When the ACP is correctly recognized by the adenylation enzyme, the thiol group of the cysteine residue functions as a nucleophile, reacting with the α-bromoacetyl group of *crypto*-ACP to form a cross-linked complex. The crystal structure of the HitB–HitD cross-linked complex revealed the interface between these proteins, illustrating their favorable interaction. This unique protein–protein interaction (PPI) appears to be a key factor for the selective transfer of the β-amino acid to the cognate ACP to produce β-aminoacyl-ACP in selective biosynthesis.

It is also intriguing how VinM selectively recognizes 3AIB-VinL (**17**) by distinguishing it from the holo form of VinL (**15**) and 3-MeAsp-VinL (**16**). To address the importance of the length of the β-aminoacyl moiety in 3AIB-VinL (**17**), we prepared longer versions of the α-bromoacetamide pantetheine mimic by replacing the 1,2-ethylenediamine moiety with 1,3-propanediamine, 1,4-propanediamine, or 1,6-hexanediamine, yielding C3Br (**27**), C4Br (**28**), and C6Br (**29**) (Fig. 8) [50]. When we used C6Br-crypto-VinL for the cross-linking reaction with the VinM cysteine mutant, the cross-linked complex was efficiently produced. The crystal structure of the VinM–VinL complex with C6Br (**29**) illustrated the PPI between VinM and VinL. Interestingly, the pantoate moiety of C6Br is bent, and the dimethyl group interacts with a tyrosine residue that is conserved among the VinM family of adenylation enzymes. Therefore, VinM appears to recognize the pantoate moiety in 3AIB-VinL (**17**) and adjusts the location of the nucleophilic β-amino group for the amide bond-forming reaction to give l-Ala-3AIB-VinL (**18**). This result indicates that the phosphopantetheinyl group is not only a linker that connects the ACP and the acyl group, but is also selectively recognized by enzymes that transfer acyl groups.

The cross-linked complex between the dipeptidyltransferase VinK and VinL, using bismaleimide and C2Br (**25**) as cross-linkers, revealed an appropriate PPI between these proteins [51, 52]. Furthermore, VinK has a large cavity at its active site, which accommodates the dipeptide moiety of l-Ala-3AIB-VinL (**18**). Therefore, VinK seems to distinguish l-Ala-3AIB-VinL (**18**) from 3-MeAsp-VinL (**16**) and 3AIB-VinL (**17**) and selectively transfers the dipeptidyl moiety to the loading ACP domain. Overall, these studies allowed us to elucidate the detailed mechanism of the biosynthesis of vicenistatin.

## Biosynthesis of β-amino acids in macrolactam antibiotics: unique mechanisms in each pathway

**Fig. 9:**
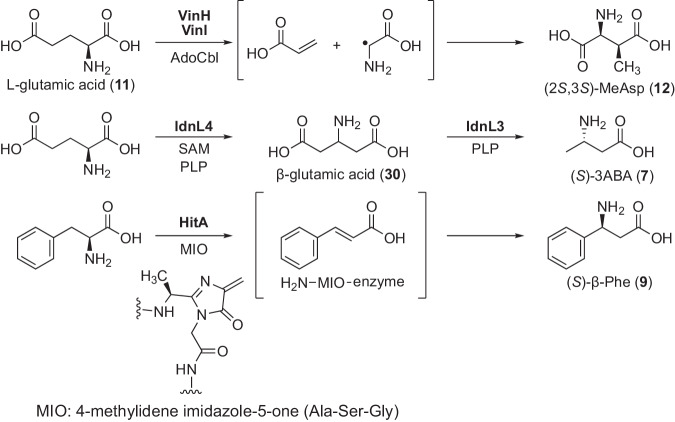
Biosynthesis of β-amino acids

β-Amino acids, being non-proteinogenic amino acids, are biosynthesized in each macrolactam producer strain. In vicenistatin biosynthesis, (2*S*,3*S*)-3-MeAsp (**12**) appears to be biosynthesized from l-glutamic acid (**11**) through the action of AdoCbl and a PLP-dependent glutamate mutase [23], comprising the E subunit VinI and the S subunit VinH (Fig. 9) [24]. Disruption of the *vinI* gene abolishes vicenistatin production, which confirms the involvement of the glutamate mutase E subunit VinI in the formation of (2*S*,3*S*)-3-MeAsp (**12**). However, mutants with the disrupted *vinI* gene produced 18-desmethylvicesistain, supposedly biosynthesized from l-Asp (**21**) [53]. Since VinN recognizes l-Asp (**21**) with low efficiency [25], downstream enzymes may accommodate the l-Asp-derived intermediates to biosynthesize 18-desmethylvicenistatin.

In incednine biosynthesis, (*S*)-3-aminobutyric acid (3ABA, **7**) is biosynthesized from l-glutamic acid (**11**), as determined by feeding experiments [54, 55]. Interestingly, incorporation studies revealed that β-glutamate (**30**) is the biosynthetic intermediate. A PLP-dependent decarboxylase, IdnL3, was found to recognize β-glutamate (**30**) and catalyze decarboxylation to give (*S*)-3ABA (**7**) (Fig. 9) [32]. In the incednine BGC, a radical *S*-adenosyl-l-methionine-dependent enzyme, IdnL4, is encoded and is likely responsible for rearranging the α-amino group of l-Glu (**11**) to the β-position to give β-glutamate (**30**) (functioning as a PLP-dependent glutamate 2,3-aminomutase [56]). We disrupted the *idnL4* gene to confirm that *idnL4* is involved in the biosynthesis of 3ABA (**7**) [57], although the expected enzymatic activity of IdnL4 has not been observed in vitro. Furthermore, we demonstrated mutasynthesis of incednine analogs using the Δ*idnL4* strain with 3-aminopentanoic acid (3APA) as an alternative substrate for IdnL1. Since IdnL1 shows strict substrate specificity against β-amino acid substrates, only 3ABA (7) and 3APA were incorporated into the pathway to produce natural incednine and 28-methylincednine [57].

**Fig. 10:**
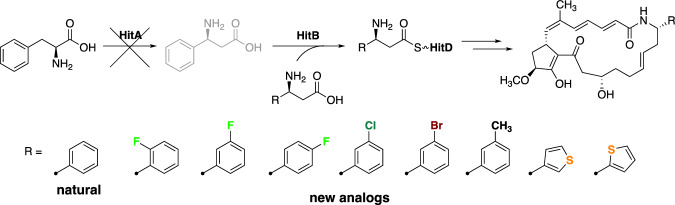
Mutasynthesis of hitachimycin analogs

In hitachimycin biosynthesis, (*S*)-β-Phe (**9**) appears to be biosynthesized from l-α-phenylalanine by the action of phenylalanine aminomutase HitA (Fig. 9). The enzymatic activity of HitA has been confirmed [34]. Additionally, the *hitA* gene was disrupted to verify its involvement in the biosynthesis of hitachimycin [34]. Supplementation of (*S*)-β-Phe (**9**) into the Δ*hitA* strain was required to recover hitachimycin production. Interestingly, HitB shows tolerant substrate specificity, recognizing several (*S*)-β-Phe derivatives [46]. Thus, mutasynthesis of hitachimycin analogs was conducted with the Δ*hitA* strain [46] (Fig. 10). In mutasynthesis, it was clarified that HitB functions as a gatekeeper in the biosynthesis, as most (*S*)-β-Phe derivatives recognized by HitB were incorporated into the pathway, yielding the corresponding hitachimycin analogs.

**Fig. 11:**
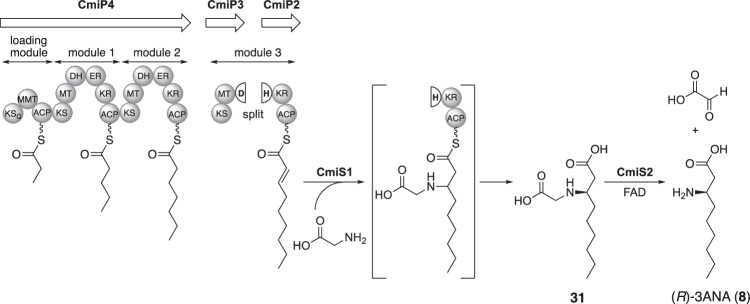
Biosynthesis of (*R*)-3-aminononanoic acid in cremimycin biosynthesis

In cremimycin biosynthesis, 3-aminononanoic acid (3ANA, **8**) is biosynthesized via the polyketide pathway, as determined by feeding experiments [58]. In the cremimycin BGC, two sets of type I PKSs, one for 3ANA biosynthesis and one for macrolactam biosynthesis, are encoded [33]. The PKS for 3ANA biosynthesis consists of CmiP4, CmiP3, and CmiP2. Interestingly, a DH domain is split into CmiP3 and CmiP2 (Fig. 11). Furthermore, there is no TE domain in the PKS to release the polyketide chain for 3ANA (**8**) biosynthesis. A split DH domain was artificially connected to verify the dehydration activity. As a result, the split DH domain catalyzed the dehydration of (*R*)-2-hydroxynonanoyl-*N*-acetylcysteamine (NAC) thioester to yield (*E*)-2-nonenoate [59]. Additionally, a unique dual-functional thioesterase, CmiS1, was found to catalyze the Michael addition of glycine to the β-position of (*E*)-2-nonenoate and the subsequent hydrolysis of the thioester to give *N*-carboxymethyl-3-aminononanoic acid (**31**) [33]. Crystal structure analysis of a CmiS1 homolog SAV606 revealed a unique substrate recognition and reaction mechanism [60]. *N*-Carboxymethyl-3-aminononanoic acid (**31**) is then recognized by a flavin adenine dinucleotide (FAD)-dependent dehydrogenase CmiS2 and converted to (*R*)-3ANA (**8**), a unique β-amino acid macrolactam starter unit in cremimycin biosynthesis [33]. CmiS2 seems to catalyze dehydrogenation, to generate an imine intermediate, and subsequent hydrolysis, to afford glyoxylic acid and (*R*)-3ANA (**8**) [61]. Interestingly, CmiS1/SAV606-type thioesterase is involved in the biosynthesis of isonitrile-containing natural products to produce *N*-carboxymethyl-3-amino-fatty acid [62], which is converted to a fatty acid with an isonitrile group at the β-position by the action of α-ketoglutarate-dependent non-heme iron oxygenase [63–66].

Overall, unique β-amino acids are biosynthesized by pathway-specific enzymes in each macrolactam producer strain to supply the appropriate starter units.

## Unique mechanisms of post-PKS modification

**Fig. 12:**
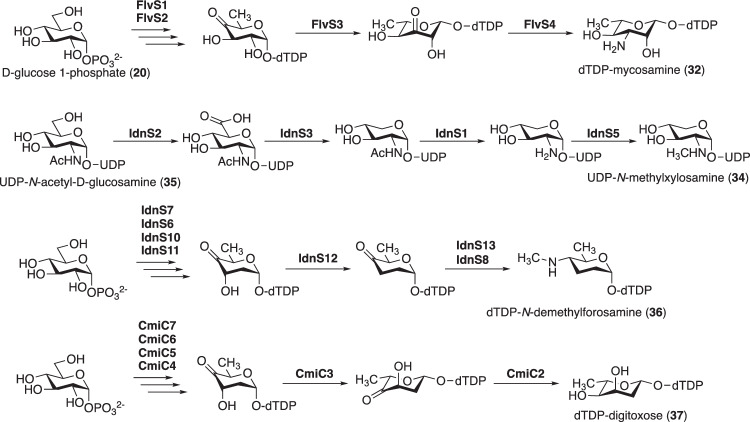
Putative biosynthetic pathways of NDP-deoxy(amino)sugars

**Fig. 13:**
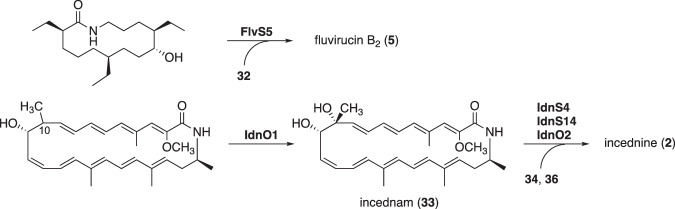
Putative glycosylation reaction in the biosynthesis of fluvirucin B_2_ and incednine

In vicenistatin biosynthesis, the aglycone vicenilactam (**14**) undergoes glycosylation with dTDP-vicenisamine (**19**), catalyzed by the glycosyltransferase VinC, to complete the biosynthesis (Fig. 6) [24]. This post-PKS modification is crucial for the biological activity of vicenistatin. The unique glycosyl donor dTDP-vicenisamine (**19**) is biosynthesized by unique deoxyaminosugar biosynthetic enzymes, as described earlier [19]. A similar scenario is also seen in fluvirucin B_2_ biosynthesis (Figs. 12 and 13) [35]. The glycosylation of the aglycon of fluvirucin B_2_ with dTDP-l-mycosamine (**32**) by FlvS5 is essential to complete the biosynthesis. dTDP-l-mycosamine (**32**) is likely biosynthesized by a set of NDP-deoxyaminosugar biosynthetic enzymes: dTDP-glucose synthase FlvS1, dTDP-glucose 4,6-dehydratase FivS2, dTDP-4-keto-6-deoxyglucose 3,5-epimerase FlvS3, and dTDP-sugar aminotransferase FlvS4 (Fig. 12). The unique combinations of aglycones and deoxyaminosugars are significant in the biosynthesis of specific macrolactam compounds with distinct biological activities.

In incednine biosynthesis, the aglycone incednam (**33**) appears to undergo glycosylation with two unique sugars, *N*-methylxylosamine and *N*-demethylforosamine, presumably by glycosyltransferases IdnS4 and IdnS14, and possibly with the assistance of an auxiliary cytochrome P450-type protein, IdnO2 (Fig. 13) [32]. UDP-*N*-methylxylosamine (**34**) is likely biosynthesized from UDP-*N*-acetyl-d-glucosamine (GlcNAc, **35**) through the action of UDP-GlcNAc 6’-dehydrogenase IdnS2, UDP-*N*-acetylxylosamine (XylNAc) synthase IdnS3, UDP-XylNAc deacetylase IdnS1, and *N*-methyltransferase IdnS5 (Fig. 12). dTDP-*N*-demethylforosamine (**36**) is presumably biosynthesized from d-glucose 1-phosphate (**20**) through the sequential action of the following enzymes: dTDP-glucose synthase IdnS7, dTDP-glucose 4,6-dehydratase IdnS6, dTDP-4-keto-6-deoxyglucose 2,3-dehydratase IdnS10, dTDP-3,4-diketo-2,6-dideoxyglucose 3-ketoreductase IdnS11, dTDP-4-keto-2,6-dideoxyglucose 3,4-dehydratase IdnS12, aminotransferase IdnS13, and *N*-methyltransferase IdnS8 (Fig. 12). Incednam (**33**) is unlikely to be the macrolactam that is initially formed by PKSs IdnP1, IdnP2, IdnP3, IdnP4, and IdnP5, as it contains a hydroxy group at the C-10 position. This hydroxy group is presumably introduced by a cytochrome P450 monooxygenase, IdnO1, during post-PKS modification, although the exact point at which it does in the enzymatic pathway remains unclear.

**Fig. 14:**
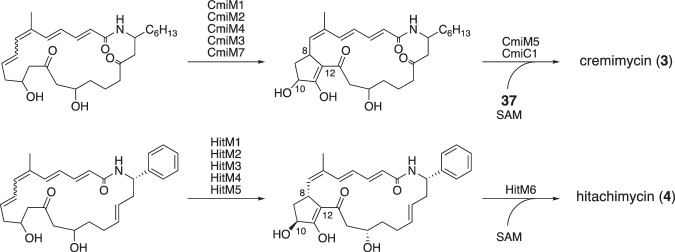
Putative post-PKS modification in cremimycin and hitachimycin biosynthesis

Cremimycin (**3**) and hitachimycin (**4**) share a common bicyclic polyketide skeleton, with the C-8 and C-12 positions presumably linked by post-PKS modification enzymes (Fig. 14). Comparison of the BGCs of cremimycin and hitachimycin suggests the involvement of five homologous enzymes (two nicotinamide adenine dinucleotide cofactor-dependent oxidoreductases (CmiM1/HitM1 and CmiM3/HitM4), a cytochrome P450 (CmiM4/HitM3), and two putative sugar phosphate isomerase/epimerase type enzymes (CmiM2/HitM2 and CmiM7/HitM5)) in the formation of the common bicyclic structure [33, 34]. Functional analysis of these enzymes will pave the way to understanding the unique mechanism of polyketide backbone modification.

In cremimycin biosynthesis, the glycosylation reaction likely occurs after the formation of the bicyclic structure; this is because the hydroxy group at C10 of cremimycin appears to be introduced during the modification of polyketide backbone, as determined by the domain structures of the PKSs. Glycosyltransferase CmiM5 is likely responsible for glycosylation with dTDP-digitoxose (**37**), which is presumably biosynthesized by the following enzymes: dTDP-glucose synthase CmiC7, dTDP-glucose 4,6-dehydratase CmiC6, dTDP-4-keto-6-deoxyglucose 2,3-dehydratase CmiC5, dTDP-3,4-diketo-2,6-dideoxyglucose 3-ketoreductase CmiC4, dTDP-4-keto-2,6-dideoxyglucose 3,5-epimerase CmiC3, and dTDP-4-keto-2,6-dideoxyhexose 4-ketoreductase CmiC2 (Fig. 12) [33]. The final step in the biosynthesis of cremimycin is likely *O*-methylation of the digitoxose moiety by methyltransferase CmiC1.

In hitachimycin biosynthesis, the C10-OH group is anticipated to be *O*-methylated by the methyltransferase HitM6 in the final step of post-PKS modification.

## Future perspectives

The next challenge for the field is to build upon the accumulated knowledge of the biosynthetic mechanisms of β-amino acid macrolactam antibiotics to engineer designer macrolactam compounds. As mentioned earlier, we have successfully produced hitachimycin analogs through mutasynthesis with β-Phe derivatives. However, the scope of the derivatives was limited because the β-amino acid-selective adenylation enzyme HitB did not accept most *o*- and *p*-substituted β-Phe derivatives, with the exception of small fluorine-substituted analogs. To address this limitation, the active site of HitB must be engineered to accommodate *o*- and *p*-substituted β-Phe derivatives, which would enable the production of a diverse range of hitachimycin analogs. Consequently, the engineering of adenylation enzymes to broaden their substrate scope is a crucial next step.

Moreover, β-amino acid-selective-adenylation enzymes can be exchanged to alter the β-amino acid starter units, leading to the production of new macrolactam compounds. Given the unique PPI revealed by the HitB–HitD cross-linked complex, it is essential to confirm whether the exchanged β-amino acid-selective adenylation enzyme can interact with different ACPs to ligate non-native β-amino acids. To overcome potential challenges in PPIs, designing specific interactions between each adenylation enzyme and the ACP may be necessary to introduce unnatural β-amino acids into the pathway, facilitating the production of designer macrolactams. The engineering of type I PKSs, including the exchange, introduction, and deletion of domains, can also be applied to the biosynthetic machinery of macrolactam antibiotics.

This biosynthetic journey, starting from the discovery of vicenistatin, has spanned three decades. Challenges still lie ahead as we strive to create novel macrolactam compounds with improved biological activities.
